# Chloral and Strychnine

**Published:** 1871-04

**Authors:** 


Chloral and Strychnine.—Dr. Liebreich establishes a comparison between these two substances, in the *Berliner Kl. Woch.*, No. 43, 1870; and finds that they neutralise each other. As the symptoms of poisoning by strychnine are very similar to those of tetanus, chloral would be a sovereign remedy for the latter, if its effects could be made to extend longer than the pathological irritation (actual nature unknown) which gives rise to tetanus. Great improvement is, however, obtained by using the hydrate, as it seems to change the forms of the complaint from the acute to the chronic, a change very likely to be followed by a cure. The salt should be given in the forms of enema, and the dose may vary from 37 to 75 grains. Full doses should be given at once. Hypodermic injections are only exceptionally to be used; but the author states that his injections with the platino-irridium syringe never were followed by abscess.—*Lancet.*

